# Editorial: Insights on plant-associated microorganisms: diversity, systematics and genomics

**DOI:** 10.3389/fmicb.2023.1323823

**Published:** 2023-10-31

**Authors:** James T. Tambong, Guillaume J. Bilodeau, Mamadou L. Fall

**Affiliations:** ^1^Ottawa Research and Development Centre, Agriculture and Agri-Food Canada (AAFC), Ottawa, ON, Canada; ^2^Ottawa Plant Laboratory, Canadian Food Inspection Agency, Ottawa, ON, Canada; ^3^Saint-Jean-sur-Richelieu Research and Development Centre, Agriculture and Agri-Food Canada (AAFC), Saint-Jean-sur-Richelieu, QC, Canada

**Keywords:** plant-associated microorganisms, phylogeny, phylogenomics, bacterial diversity, taxonomy, fungal diversity, viral diversity

## 1. Introduction

The purpose of this Research Topic call was to provide insights into community diversity and complex associations or interactions on plant-associated microorganisms: bacteria, fungi, and viruses. Twenty-one papers addressing this key premise of the Research Topic were published into four loosely defined categories: diversity, taxonomy, and detection of plant pathogens; soil and rhizosphere microbial communities; seed and ephiphytic microbiome; and beneficial bacteria. Eleven of the 21 papers addressed issues related to a better understanding of bacterial and fungal plant pathogens as well as their accurate taxonomic identification and detection. Six articles explored microbial communities in diverse environments including areas contaminated with antimony and arsenic. Two articles addressed the potential applications of bacteria, while another two centered around the intricate world of seed and epiphytic microbiomes. This editorial provides a comprehensive summary of each paper within these four distinct categories, offering a detailed overview of the valuable research conducted in these fields.

## 2. Summary of published papers

This section is subdivided into the four categories of articles.

### 2.1. Diversity, taxonomy, and detection of plant pathogens

The papers in this subsection addressed fungal (seven), bacterial (three), and viral (one) diseases or pathogens, providing insights on their taxonomy, pathogenicity/virulence factors or an innovative detection system.

Lu et al. isolated eight bacterial strains from tobacco fields and used polyphasic method consisting of whole genome sequence comparisons, and phenotypic and chemotaxonomic characterizations to delineate the strains to species-level. All the strains were taxonomically affiliated to the genus *Ralstonia* based on 16S rRNA gene analysis. Genome-based parameters and phylogenomic analysis provided insights in species-level affiliation with one strain, 22TCCZM03-6, assigned to a known species, *Ralstonia wenshanensis*. The seven remaining strains could not be assigned to known *Ralstonia* species, and based on the data these were described into three new species, *Ralstonia soli* (21MJYT02-11), *Ralstonia mojiangensis* (21MJYT02-10, 21LDWP02-16, 22TCJT01-1, 22TCCZM01-4, and 22TCJT01-2), and *Ralstonia chuxiongensis* (21YRMH01-3 and 26). Lu et al. also performed gene content analysis of these novel *Ralstonia species*.

Myers et al. hypothesized that genes involved in necrosis in *Allium* spp. and confer thiosulfate tolerance co-occur (associate) as these are co-beneficial traits for the plant's survival in the niche. Also, they postulated that genes that result in the production of toxic byproducts or have redundant functions should dissociate to minimize their co-expressive effects on the bacterial fitness. As such Myers et al. analyzed the interactions of accessory genes in a pangenome of 92 *Pantoea ananatis* genomes in view of understanding the association and dissociation patterns of some of the virulence genes. Pathogenic phenotyping of the 92 *P. ananatis* strains revealed variability in aggressiveness levels on *Allium* spp. Genome-wide analysis identified, 835 pathogenesis-related genes in *P. ananatis* against *Allium fistulosum* × *Allium cepa* and 243 genes were associated with the infectivity of *Allium porrum*. Gene-pair coincidence analyses identified 165 individual genes classified into 39 significant gene-pair association components and 255 genes in 50 significant dissociation components. The authors then performed comparative genomics analysis on five *P. ananatis* strains that showed differential pathogenicity on *A. porrum* or *A. fistulosum* × *A. cepa* and identified candidate genes that explained the difference in virulence. The authors found a putative type III secretion system, and several other genes and showed by mutational analysis that the *pep*M gene in the HiVir cluster is important than the same gene within the pgb cluster for pathogenicity of *A. fistulosum* × *A. cepa* and *A. porrum* by *P. ananatis*.

Agarwal et al. analyzed the pangenome of 1,910 *Xanthomonas* strains to assess genus-wide genetic diversity and generate a genus-wide phylogeny. Also, the Agarwal et al. reclassified the *Xanthomonas* strains into species clusters using a phylogenetic framework and identified sampling gaps based on via rarefaction analyses. Other results of Agarwal et al. show the distribution and evolution of the T3SSs and T3SEs as these play important roles in host specificity and further analysis identified three structurally and evolutionarily distinct T3SS pathogenicity islands due probably to three independent acquisition events. Based on the recombination data, Agarwal et al. concluded that recombination is frequent among members of the genus *Xanthomonas*.

Batarseh et al. examined the comparative genomics of the genus *Liberibacter* and concluded high genomic content diversity as well as positive selection based on 52 genome sequences from six species. Ca. *Liberibacter asiaticus* had the highest number of genome sequences of 36. The authors used average nucleotide identity values to confirm the taxonomic positions of the strains within and between species. The authors also investigated the patterns of how the accessory genes evolved by comparing the phylogenies of the core genes to that of the accessory genes showing a similar evolutionary pattern. Also, Batarseh et al. measured the selection patterns and identified genes showing positive selection and concluded that these might be involved in pathogenicity and virulence but using the global test, the authors showed that the core genes are in selective constraints.

Javaran et al. developed and validated a new diagnostic tool (NanoViromics) for rapid detection of dsRNA plant viruses and viroids using Oxford Nanopore sequencing technology in infected grapevines. The long-read Nanopore sequence data were analyzed using two bioinformatic workflows: Centrifuge-Recentrifuge (Cent&rec) and DIAMOND-MEGAN (DIA&MEG) and the results compared. Also, parallel Illumina MiSeq sequencing of short reads were generated and processed in two ways:(a) the reads were *de novo* assembled using Lazypipe pipeline followed by taxonomic classification using Centrifuge and then ReCentrifuge used for comparative analysis, and (b) DIAMOND-MEGAN workflow direct analysis. The direct-cDNA sequencing from dsRNA (dsRNAcD) outscored the rRNA-depleted total RNA (rdTotalRNA) method for detection of low plant virus titers. Both workflow classifiers outputted similar viral detection and taxonomic results. Javaran et al. concluded that dsRNAcD sequencing is a reliable and cost-effective tool for detection of plant viruses.

Gogoi et al. sequenced and analyzed the whole genome sequences of five *Phytophthora cactorum* strains of the two known pathotypes causing the crown rot on rhizomes or the leather rot on the fruits of strawberry. Gogoi et al. generated genome sequences by *de novo* assembly of highly and low virulent crown rot strains as well as three leather rot strains of sizes 66.4–67.6 Mb with total predicated complete genes ranging 17,286–17,398. Phylogenomic comparison of the strains sequenced by Gogoi et al. showed the *Ph. cactorum* strains to be highly similar but distinct from five other *Phytophthora* species. A better phylogenomic resolution was obtained when Gogoi et al. analyzed only the *Ph. cactorum* strains showing distinct patterns between the crown and leather rot pathogens. Gogoi et al. employed comparative genomics which revealed genes with potential involvement in pathogenesis and recommend functional studies for these candidate pathogenicity determinants.

Yang et al. generated a complete whole genome sequence of *Verticillium dahliae* strain VD991 using Oxford Nanopore technology and used the genome for screening and validation of genes involved in pathogenicity. The genome size is 35.77 Mb. Genome-based collinearity analysis of the genome sequence by Yang et al. show that the genomes of *Verticillium alfalfae* and *Plectosphaerella cucumerina* partially overlapped with that of *V. dahliae*, an indication of whole genome duplication. Yang et al. complemented this genome data with transcriptomic data from *V. dahliae* and three cotton varieties with different resistance levels to investigate possible gene interactions. The analysis of the transcriptomic data identified a network of 19 hub genes with the potential to interact with cotton genes. Yang et al.'s data show that the highest number of differentially expressed genes (DEGs) between the susceptible (JM11) and the resistant (ZZM2) cotton varieties occurred at 6 h post-inoculation and showed less down-regulated DEGs than up-regulated DEGs. The studied identified many pathways and genes that might be involved in cotton disease resistance and pathogenicity of *V. dahliae*.

Harish et al. characterized 60 strains of *Fusarium* species causing the post-flowering stalk rot on maize based on morphology, pathogenicity and molecular techniques. Harish et al. observed that the *Fusarium* strains showed high variability in cultural traits such as colony coloration, mycelial branching, and conidial size and shape; and the majority of the isolates had a white to dirty white colony color. Based on pathogenic effects on maize seed germination, eight strains were categorized to be of low virulence, 20 moderately virulent and 27 virulent isolates. The authors report significant differences in root length and seedling vigor between *Fusarium*-treated and untreated control maize seeds. Harish et al. molecularly identified the 10 most virulent strains, based on partial sequences of the translation elongation factor 1a (TEF-1a), to taxonomically belong to *Fusarium acutatum, Fusarium verticillioides*, and *Fusarium andiyazi*.

Tu et al. examined *in planta*-expressed genes in *Fusarium graminearum* during the infection of susceptible and resistant wheat varieties using comparative transcriptomics. Tu et al. showed that host genotypes regulate the gene expression patterns of *F. graminearum*. Spikes were inoculated with *F. graminearum* and total RNA extracted for samples at different time points. Tu et al. indicate a dynamic distribution of differentially expressed genes (DEGs) of *F. graminearum in planta* in the susceptible and resistant wheat varieties over the time-points with 533 DEGs identified. Also, their data on functional enrichment of the DEGs identified in *F. graminearum* reveal the involvement of different infection mechanisms in resistant and susceptible wheat varieties. Also, the Tu et al. observed dynamically expressed putative effectors in the two wheat genotypes. The reliability of the DEGs generated using RNA-seq were confirmed by qRT-PCR.

Achilonu et al. used random amplified microsatellites (RAMS) with primers CCA_5_ and CGA_5_ to cluster 364 South African isolates of *Alternaria alternata* (the causal agent of the pecan black spot disease) into two major distinct clades and 10 sub-clades. Achilonu et al. report a low genetic diversity within the *A. alternata* populations isolated from the eight major pecan-producing provinces of South Africa. The authors attribute the low diversity potentially to high gene flow within the populations. STRUCTURE analysis of the generated alleles did not show even distribution of genotypes and as such no distinct geographic or locational relationship was observed.

Dettman et al. molecularly characterized 558 strains of *Alternaria* section *Alternaria* isolated from 64 host plant genera in 12 countries. The authors used two section *Alternaria*-specific markers (*ASA-10* and *ASA-19*) and the second largest subunit of RNA polymerase II (*rpb2*) gene in the study. Canadian isolates made up 57.4% of the total population studied. Dettman et al. showed that the most common species on cereal crops in Canada are *Alternaria alternata* (83%) and *Alternaria arborescens* (10.6%). Dettman et al. report that the Canadian isolates did not cluster into geographic-specific clades as well as very low association between hosts and genetic haplotypes. The *A. arborescens* isolates formed, at least, three distinct phylogenetic lineages. Other minor lineages identified by Dettman et al. are *Alternaria gaisen* (4.5%), the longipes lineage (1.4%; *Alternaria longipes* and *Alternaria gossypina*), as well as monotypic lineages of single strain of *Alternaria burnsii* (CBS107.38) and the undescribed species, each forming a distinct lineage in phylogenetic trees generated with the ASA-10 and ASA-19 markers.

### 2.2. Soil and rhizosphere microbiome

These group of six papers examined microbial communities in soil, rhizosphere and plant root-nodules.

Bromfield et al. characterized a bacterial strain T173 isolated from a root-nodule of white sweet clover growing in Canada. The authors used polyphasic analysis including genome-based DNA-DNA hybridization and average nucleotide identity to conclude that this strain constitutes a novel *Ensifer* lineage and the name *Ensifer canadensis* is proposed. Bromfield et al. sequenced the genome using the Pacific Biosciences technology and identified six replicons consisting of a chromosome and five plasmids. One of the plasmids harbors symbiosis genes involved in nodulation and nitrogen fixation which the authors suggest could be the result of horizontal transfer from *Ensifer medicae*. Bromfield et al. also reported the unexpected presence of two single copies (out of five) of the complete ribosomal RNA operon (*rrn*) on two of the megaplasmids. Based on this and other genomic features of one of these megaplasmids (pT173e), the authors suggest it could be a “chromid”-a secondary chromosome.

Mi et al. examined the community diversity of arbuscular mycorrhizal fungi (AMF) in soils contaminated with antimony (Sb) and arsenic (As) using morphological and molecular techniques. The authors collected soil samples from two different areas with different contamination levels of the heavy metals. Mi et al. noted differences on how the AMF colonize the *Artemisia argyi* (more external hyphae with vesicles at the tips) relative to numerous internal hyphae produced on *Rumex acetosa* and *Carpesium abrotanoides* roots. AMF colonization rate was heavily influenced by plant species. Their molecular results based on 18S rRNA high throughput sequencing detected 268 AMF OTUs with 88.27% belonging to the *Glomeraceae*; and show that As and its interaction with P had a stronger effect in the reduction of soil AMF richness and diversity than Sb.

Swiatczak et al. report that the application of *Pseudomonas sivasensis* strain 2RO45 in the canola rhizosphere alters the microbial structure and functioning communities. The authors used high throughput sequencing of the V3–V4 hypervariable 16S rRNA and ITS2 to assess the communities. Their results show that the application of strain 2RO45 did not affect the overall microbial diversity but altered the taxa proportions of bacteria and fungi. The authors showed changes from the abundant *Streptomyces*, at an initial time of application, to bacterial families such as *Comamonadaceae* and *Vicinamibacteraceae* 22 days after application. They observed a similar pattern from *Cyphellophora vermispora* (day 0) to a fungal family of *Nectriaceae*, genus *Exophiala* and species *Mortierella minutissima*.

Nanetti et al. hypothesized that the composition and/or the diversity of specific microbial communities associated with *Vitis vinifera* at the soil–root interface can be used to define and protect a wine “Protected Designation of Origin” (PDO) area. They collected grapevine roots and soil samples in June and November from three distinct vineyards: a non-PDO (conventional farming), a PDO area under conventional farming and a PDO area under organic farming. Nanetti et al. performed high throughput sequencing and analysis of the V3–V4 region of the 16S rRNA to find that some bacterial genera were more abundant in a particular vineyard irrespective of the season in bulk soils. Also, they detected rhizospheric bacterial genera that discriminated PDO-associated from non-PDO communities that are independent of management type, season or farming site.

Hossain et al. investigated the microbial communities in different sized nodules in peanut plants inoculated with host-specific rhizobia under field conditions. They reported that the inoculated peanuts formed regular sized (large) and small nodules with the former having red interior color and suggesting active nitrogen fixation. The colorations of the small nodules, however, varied and seemed to show limited fixation activity even though the *nif* H gene was amplified from the two types of nodules. The bacterial diversity in the two types of nodules were significantly different based on amplified sequence variants (ASVs). The ASVs in both nodule types were largely Proteobacteria with the big nodules showing 99.96% (0.03% Actinobacteria) while the small ones had only 68.67% (31.32% other bacterial taxa). The authors' results at the genus-level showed the top taxa in the big nodule samples had primarily *Bradyrhizobium* while the small nodules contained other taxa. Based on the functional analysis, the authors suggest that the diverse microbial communities in the small nodules beside the Proteobacteria, could play other biological roles such as the production of secondary metabolites including phytohormones, antibiotics and others.

Li et al. compared the microbial diversity and structure in the rhizospheres of straight and twisted trunk types of *Pinus yunnanensis* using 16S rRNA and ITS metagenomics. Li et al. showed significant differences in available phosphorus between the two trunk types and indicated that available potassium had a significant effect on the fungal community. Their results show a statistically higher relative abundance of Proteobacteria in the rhizosphere soil of the twisted than the straight trunk. At the genus-level, Li et al. found the phylum *Chloroflexi* to be relatively low in twisted compared to straight trunks. *Basidiomycota* and *Ascomycota* were the dominant fungal phyla in both trunk types but were not statistically different. At the genus level, the relative abundances of *Penicillium* and *Fusicolla*, potential plant pathogens, were higher in the twisted trunk samples while *Penicillium nodositatum* was significantly higher at the species level. Li et al. also identified bacterial and fungal biomarkers that were differentially abundant in the twisted or straight trunk type.

### 2.3. Plant epiphytic and seed microbiomes

Zhou et al. examined the epiphytic microbial communities of wild soybeans across China. The authors collected foliage and seed samples of wild soybeans and identified the plant genotypes using simple sequence repeats (SSR) technology. SSR method determined that the different eight locations harbor distinct genotypes that could be clustered into three major groups. The composition and diversity of the bacterial and fungal communities were examined by high throughput sequencing and analyses of the 16S rRNA and internal transcribed spacers (ITS), respectively. Their results identify Proteobacteria as the dominant foliar bacterial community while *Ascomycota* and *Basidiomycota* were the dominating fungal taxa. The results of Zhou et al. showed, also, that the high abundance core foliar microbiota were present in all wild soybean samples, irrespective of genotype and/or environmental conditions.

Malacrinò et al. studied the influence of six cereal crop species on the diversity and structure of their seed fungal microbiomes by high throughput sequencing of the ITS2 region. The authors analyzed the 65 samples to identify 242 fungal Amplicon Sequence Variants (ASVs). The authors point that the seed fungal diversity and structure are influenced by the cereal species but these differences are mainly driven two species, *Avena sativa* and *Hordeum vulgare*. Malacrinò et al. did not find significant evidence of phylosymbiosis—relationship between microbial community and the host. The authors, however, showed significant differences in fungal community structure of different cultivars of *Triticum aestivum* and *Triticum turgidum*.

### 2.4. Beneficial bacteria

Jiang et al. isolated a novel endophytic strain AK-PDB1-5 from leaves of the Korean fir and identified the bacterium based on 16S rRNA, whole-genome sequences, biochemical and biophysical data. The results suggest that this strain is a new bacterial species within the genus *Sphingomonas* and produces a yellow carotenoid pigment known as nostoxanthin. Jiang et al. also report studies with Arabidopsis seeds inoculated with the bacterium with results showing potential plant growth-promoting and salt tolerance effects induced by this strain. The authors data also show that salt tolerance effect of strain AK-PDB1-5T in Arabidopsis roots is by scavenging the reactive oxygen species probably by producing the nostoxanthin carotenoid.

Xie et al. sequenced the whole-genome sequence of *Bacillus velezensis* strain YC89, a strain that was isolated from infected sugarcane plants. The whole genome sequence was generated using PacBio technology. The authors mined complete genome sequence for genes involved in secondary metabolite production leading to the discovery of 12 clusters as well as eight genes related to indole acetic acid (IAA) biosynthesis which is key to plant growth-promotion including resistance inducer. The authors also detected the presence of siderophores, cellulase activity and phosphate solubilization. Finally, Xie et al. also report the biocontrol of the red rot disease of sugarcane by *B. velezensis* strain YC89 in greenhouse plant assays. Xie et al. concluded that strain YC89 is a potential potent biological control agent as well as a biofertilizer.

## 3. Conclusion

The publication of these 21 articles with over 250 eprint-pages is testament to the success of this Research Topic. In reviewing the published articles, it could be noted that the bulk of the articles addressed the “genomic and metagenomic studies of uncultured plant-associated bacteria and fungi” focus of the Research Topic. This, perhaps, is indicating significant interest and/or shift of microbiological work to prioritizing the use of culture-independent approaches. This might be partly fueled by recent advances in high throughput sequencing technologies coupled with the scientists' zeal to study yet unculturable microorganisms which constitute the microbial silent majority. Also, it could be inferred from reviewing the articles that culture-dependent articles addressed taxonomic/systematics studies of plant-associated beneficial and pathogenic bacteria and fungi with high reliance on whole genome sequencing and analysis. Overall, these articles have generated lots of scientific data essential for our understanding of some key aspects of plant-associated microorganisms in agriculture and forestry. Based on the high visibility with over 27,000 views and 4,417 downloads of these articles, at the time of the preparation of this editorial, it is clear that the information provided in these articles is of interest to other scientists.

## Author contributions

JT: Writing—original draft, Writing—review & editing. GB: Writing—review & editing. MF: Writing—review & editing.

